# DNA Hairpins as Temperature Switches, Thermometers and Ionic Detectors

**DOI:** 10.3390/s130505937

**Published:** 2013-05-10

**Authors:** Anette Thyssen Jonstrup, Jacob Fredsøe, Anni Hangaard Andersen

**Affiliations:** Laboratory of Genome Research, Department of Molecular Biology and Genetics, Aarhus University, C.F. Moellers Allè 3, Building 1130, 8000 Aarhus C, Denmark; E-Mails: jo@rosborg-gym.dk (A.T.J.); jcf@mb.au.dk (J.F.)

**Keywords:** temperature sensing, DNA hairpin, fluorescence readout, temperature switch, ionic detector

## Abstract

Temperature is of major importance in most branches of science and technology as well as in everyday life, and with the miniaturization of electronic devices and the increasing ability to make research into small-scale systems, a specific need for very small thermostats and thermometers has been created. Here we describe how DNA molecules can be used as nanoscale sensors to meet these requirements. We illustrate how the hybridization kinetics between bases in DNA molecules combined with conformational changes of the DNA backbone can be exploited in the construction of simple but versatile temperature switches and thermometers, which can be built into electronic systems. DNA based sensors are at the same time applicable as ion detectors to monitor the chemical environment of a specific system.

## 1. Introduction

DNA contains the genetic information of all living organisms and therefore has received extensive attention. Based on the self-assembly architecture and relative high physicochemical stability of DNA, researchers have found additional applications for the molecule as for instance in the construction of nano-sized networks and structures [1–6]. Besides its inherent features, the applicability of DNA is greatly enhanced by the ease of synthesizing predefined DNA sequences and amplifying DNA fragments by PCR. It can furthermore be modified in a vast number of ways chemically or by use of enzymes of various kinds, which expand the usability of the molecule even more.

DNA does not have a static structure. The hydrogen bonds between the bases in the DNA duplex can be broken and the two strands thereby separated. A rise in temperature or a decrease in the ionic strength of the aqueous solution facilitates this process of “DNA melting”. The fraction of melted DNA at a given temperature and ionic strength is a reflection of the equilibrium process between melting and re-hybridization of DNA and depends on the number of hydrogen bonds between the DNA strands and thus the amount and composition of base pairs. By nature, the hybridization kinetics of DNA thus act as a temperature sensor [7]. In fact, nature itself makes use of a mechanism in which conformational changes in RNA are used in the cell as a sign of heat shock and successful infection of a warm-blooded host [8–10]. The power of this system has also been demonstrated by the development of artificial *in vivo* gene expression systems, which respond to temperature shifts [11,12]. Furthermore, advantage has been taken of the hybridization kinetics of DNA to generate thermo-responsive ligands from hairpin aptamers [13,14]. Here we demonstrate the applicability of DNA molecules as temperature sensors in nanoscale temperature switches and thermometers with high sensitivity in specific desired temperature ranges or as sensors monitoring the chemical environment of a system.

## 2. Experimental Section

### Fluorescent measurements

**Figure 1.**
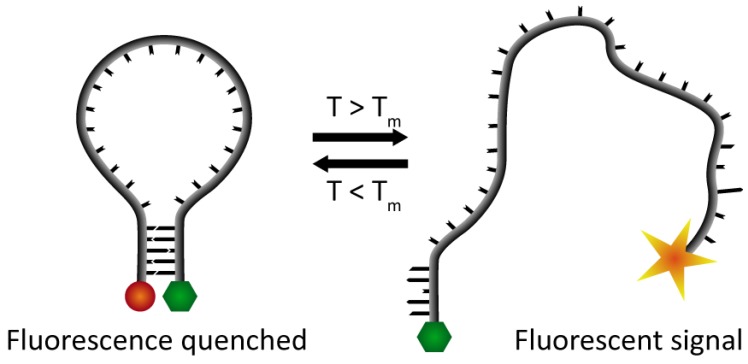
Schematic illustration of a DNA hairpin end-labeled with a donor-acceptor pair of dyes. When the DNA is in hairpin conformation, the acceptor (green) quenches the light emitted from the donor (red). Upon melting of the hairpin, the dyes are separated, and light emitted from the donor can be detected.

Fluorescent measurements were performed on an Mx4000® Multiplex Quantitative PCR System from Agilent Technologies (Santa Clara, CA, USA), which has a ramp rate of 2.2 °C/s. Oligonucleotides labeled with a donor-acceptor pair of dyes (Figure 1) (5′-end labeled with either Fam or Rox and 3′-end labeled with Black Hole Quencher 1 (BHQ1) or 2 (BHQ2), as indicated), were purchased from TAG Copenhagen A/S (Copenhagen, Denmark). The sample volume was 50 μL in all experiments.

### Hairpins and buffer conditions

**Figure 2.**
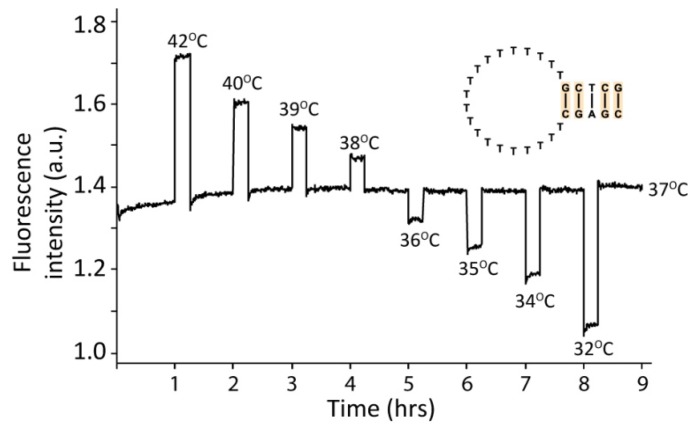
Fluorescence measurements with a dye-labeled DNA hairpin subjected to various temperatures in the range from 32 °C to 42 °C as indicated. The hairpin was first incubated at 37 °C for 60 min and then at 42 °C for 15 min. Following this was several cycles of incubation at alternately 37 °C and a test temperature for 45 min and 15 min, respectively. The hairpin sequence is as indicated.

**Figure 3.**
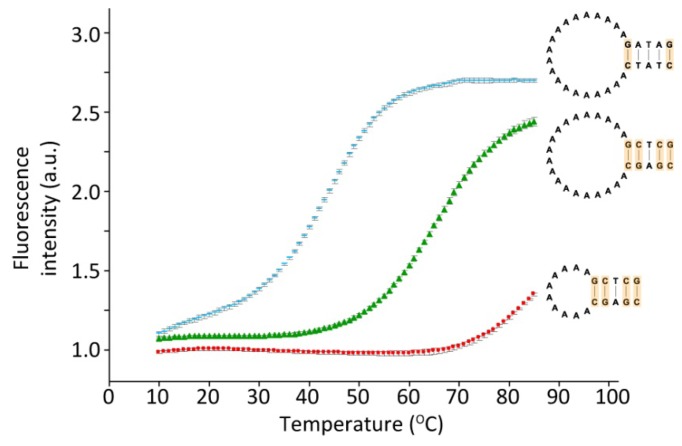
Melting curves for hairpins differing in the stem sequence and/or the loop length: Hairpins with a 20 bases long poly-A loop and a five base pair stem of which four base pairs are G-C (green), hairpins with a 20 bases long poly-A loop, where the G-C base pair content of the five base-pair stem is decreased to two (blue), or hairpins with only 10 A's in the loop and four G-C base-pairs out of five in the stem (red). Standard deviations of fluorescence values are shown as error bars. The sequence of the different hairpins is as indicated.

**Figure 4.**
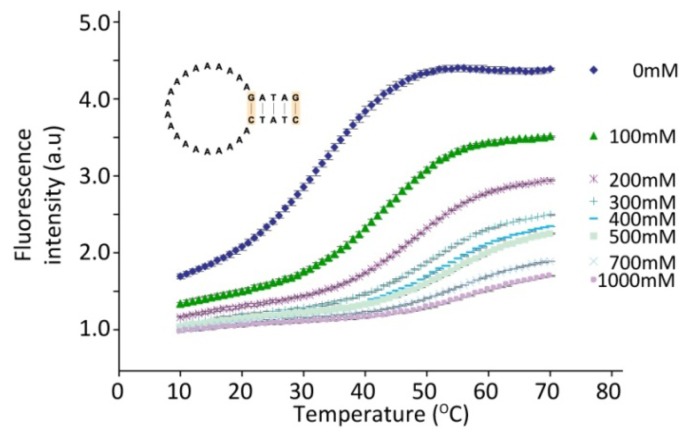
Influence of NaCl on hairpin melting. Melting curves are shown for a DNA hairpin subjected to increasing concentrations of NaCl as indicated. The hairpin has a 20 bases long poly-A loop and a five base-pair stem. Standard deviations of fluorescence values are shown as error bars. The hairpin sequence is as indicated.

The experiments were performed with 200 nM Fam5s4G-20T in 100 mM Tris-HCl, pH 8.0 (Figure 2), with 200 nM Rox5s2G-20A, 200 nM Rox5s4G-20A, and 200 nM Rox5s4G-10A in 200 mM Tris-HCl, pH 8.0, 50 mM NaCl (Figure 3), or with 200 nM Rox5s2G-20A in 100 mM Tris-HCl, pH 8 with the indicated NaCl concentrations (Figure 4).

List of hairpin sequences:

Rox5s2G-20A:
5′-ROX-CTATCAAAAAAAAAAAAAAAAAAAAGATAG-BHQ2-3′
Rox5s4G-20A:
5′-ROX-CGAGCAAAAAAAAAAAAAAAAAAAAGCTCG-BHQ2-3′
Fam5s4G-20T:
5′-FAM-CGAGCTTTTTTTTTTTTTTTTTTTTGCTCG-BHQ1-3′
Rox5s4G-10A:
5′-ROX-CGAGCAAAAAAAAAAGCTCG-BHQ2-3′

### Switch experiment

In the switch experiment presented in Figure 2 the hairpin was initially kept for 60 min at the reference temperature (37 °C) and then 15 min at each indicated temperature followed by 45 min at 37 °C. Fluorescent measurements were performed three times during the last 30 seconds of each minute. Each data-point was depicted just after the previous. It is the programmed rather than the actual temperatures, which are plotted, but they only deviated slightly from each other.

### Melting curve experiments

Generally, the programs contained the following steps: A fast denaturation step at 95 °C for 5 min (segment 1), a temperature decrease to the maximum temperature used in the particular experiment (70 °C in Figure 4 and 85 °C in Figure 3), 1 min at each degree down to 10 °C (segment 2), a temperature raise to the maximum temperature with 1 min at each degree on the way up (segment 3). Following this, seven additional segments were run through, where the temperature was alternately lowered or raised similar to segment 2 or 3, respectively (segment 4 to 10). The experiment depicted in Figure 4 only had 30 seconds at each temperature in segment 3. In segment 2–10 the fluorescence was read 3 times during the last 30 seconds at each temperature. Average fluorescent values of segment 4 to 10 were depicted in the charts. It is the programmed rather than the actual temperatures, which are plotted, but they only deviated slightly from each other. Standard deviations of fluorescence values from segment 4 to 10 are shown as error bars (one standard deviation above and one below each value).

## 3. Results and Discussion

To examine the capability of DNA molecules as sensors we have taken advantage of DNA oligonucleotides containing complementary nucleotide sequences at the ends. As a consequence of intra-strand hybridization between the complementary sequences the oligonucleotides are able to form hairpin structures, where the duplex region generated upon hybridization will form the stem of the hairpin and the nucleotides in between will form the hairpin loop. In solution equilibrium will be established between oligonucleotides in hairpin conformation and oligonucleotides, which are not self-hybridized. This equilibrium is temperature dependent. The equilibrium is changed upon alterations in the intra-strand hybridization abilities of the oligonucleotides and in the buffer condition and is characterized by the melting temperature (T_m_), which is the temperature, where half of the oligonucleotides are in the hairpin conformation. Thus, the length and base composition of the stem as well as the size and flexibility of the loop will determine, at which physico-chemical conditions the hairpins will melt and thus become single stranded. It is therefore possible to design hairpins, which sense and respond to highly specific changes in the surroundings.

To visualize the hybridization process we have used Förster Resonance Energy Transfer (FRET) between a fluorophore and a quencher located at the 5′- and 3′-end of the DNA hairpin, respectively (Figure 1). This creates so-called “molecular beacons”, which by fluorescence report the hybridization status of the DNA skeleton [7,15,16]. Thus, at temperatures below T_m_, when the DNA is in the hairpin conformation, the quencher and the fluorophore will be in close proximity of each other, and therefore the emitted light from the fluorophore will be quenched. At higher temperatures, when the DNA switches from the hairpin structure to a melted conformation the two ends of the DNA strand are separated, and the fluorescent signal from the fluorophore can be detected.

To examine the applicability of DNA hairpins as temperature sensors, a dye-labeled DNA hairpin with a five base pair stem and a 20 bases long poly-T loop was subjected to various temperatures. As seen in Figure 2 the DNA hairpin reacts significantly to small temperature changes. In this experimental setup a temperature change of one degree has a very large effect on the fluorescent signal, and thus on the conformational status of the DNA. These data demonstrate that DNA hairpins can act as highly sensitive temperature switches or thermometers.

The temperature range giving the optimal effect can be changed by altering the DNA hairpin. This can be done by modifying the length or sequence of either the hairpin stem or loop. Since the melting and hybridization reactions of hairpins are intramolecular, their equilibrium is displaced towards hybridization in comparison to intermolecular reactions. This implies that a short duplex will give rise to a much higher melting temperature when it is present as a stem in a hairpin than when it exists as an ordinary DNA duplex, obeying standard intermolecular melting-hybridization kinetics. This inherent property of hairpins sets an upper limit to the stem length within a specific detection system. However, a short stem ensures abrupt hairpin melting rather than gradual melting, and thereby inhibits formation of several intermediate conformations during melting. Like the stem also the hairpin loop will influence the melting temperature of the hairpin [17,18]. It has earlier been demonstrated that the opening rate (melting) of hairpins is independent of loop-size. However, the closing rate (hybridization) is affected, with the largest loops having the slowest closing rates most probably due to a lower probability of contact between the DNA ends with increasing loop size [19,20].

Figure 3 shows the melting profiles of three hairpins varying either in loop length having 10 or 20 A's in the loop, or in the number of hydrogen bonds in the stem, as either two or four out of five base-pairs are G-C. The melting temperature is decreased about 20 degrees when two (blue) rather than four (green) G-C base-pairs are present in the five base-pair stem. Furthermore the melting temperature is increased more than 20 degrees if the loop length is decreased from 20 to 10 A's (red). However, an increase in the loop length from 20 to 30 A's only resulted in a 5 degree reduction in the melting temperature (data not shown). A change in the loop sequence (exclusively A's, T's or C's) had no significant effect on the melting temperature (data not shown). The data thus demonstrates that the hairpins can be designed to respond to temperature changes in specific temperature ranges.

The accuracy of a DNA hairpin based sensor is optimized by increasing the steepness of the melting curve. Changing the sequence and length of the stem, the length of the loop, or totally replacing the loop with an artificial linker having the right properties will influence the abruptness of hairpin melting substantially and therefore may prove beneficial in this respect. However, a rigid loop, which supports an abrupt melting, has an inherent slow closing rate and is therefore best suited in switch applications that do not depend on a fast response time upon lowering of the temperature.

The chemical environment surrounding the DNA also influences its hybridization abilities extensively. This fact can be used to fine-tune the melting characteristics of the hairpins as illustrated in Figure 4, where the effect of an increase in the salt concentration is shown. As expected, the stabilizing effect of the positively charged Na^+^ ions in the solution on the hybridized DNA strands can be seen as an increase in the melting temperature of the hairpin with increasing salt concentration. A simple change in the buffer conditions can thus be used to modify the melting temperature of a hairpin and thereby the temperature range in which a temperature sensor shows the optimal effect. Alternatively, a hairpin can be used to directly monitor the ionic strength of the surrounding solution as demonstrated by the influence of changes in the ionic strength on the hairpin melting temperature.

The measurements conducted in the present study utilize fluorescence. The advantage of fluorescent conjugated hairpins is that multiple hairpins can be distinguished by coupling them to different fluorophores, and they can therefore be combined in the same mixture. Another advantage of the fluorescent system is that a measuring unit containing the fluorescent hairpins can be spatially separated from the readout device. However, there are disadvantages of the fluorescent system not least that the readout system likely will be cumbersome and therefore not suited for incorporation into a system, which requires a compact size. In some cases the construction of a usable miniature sensor or switch build from DNA hairpins therefore likely involves the direct coupling of the hairpins with an electrical system. The conformation of a DNA hairpin can be converted directly into an electric signal by attaching one end of the DNA hairpin to a gold surface and the other end to an electrochemical group [21–23]. In this setup, a DNA hairpin conformation allows a current between the surface electrode and the electro-active reporter, since these are in close proximity of each other. Hairpin melting, however, leads to a movement of the electro-active reporter away from the surface and turns off the current. In this system the current between the surface electrode and the electro-active reporter reflects the conformation of the DNA and thereby the temperature and ionic environment. The application of this is not least in computers and other electronic devices, where superheating is a well-known risk. Here molecular switches made up of DNA or DNA-analogue hairpins [16] or derived structures fine-tuned to react at precisely the desired temperature may be of great use as thermostats. Furthermore, the DNA sensor may prove useful to fulfill the need for ultra-small sensors when making research into small-scale systems [24–27].

## 4. Conclusions/Outlook

As demonstrated here a combination of careful hairpin design and buffer conditions allows the generation of DNA hairpins, which can function as temperature-sensitive molecular switches or thermometers within predetermined temperature ranges. This range can be increased by combining hairpins with different structures and/or in different chemical environments. Furthermore, DNA hairpins can directly be used to sense changes in the ionic environment. The fact that DNA hairpins are nanoscale sensors, which easily can be coupled to an electrical readout system, makes them highly useful as sensors in electronic devises, for which a very small size is favorable.
